# Growth Hormone Supplementation and Psychosocial Functioning to Adult Height in Turner Syndrome: A Questionnaire Study of Participants in the Canadian Randomized Trial

**DOI:** 10.3389/fendo.2019.00125

**Published:** 2019-03-13

**Authors:** Joanne F. Rovet, Guy Van Vliet

**Affiliations:** ^1^Departments of Pediatrics and Psychology, University of Toronto, Toronto, ON, Canada; ^2^Neuroscience and Mental Health Program, The Hospital for Sick Children, Toronto, ON, Canada; ^3^Department of Pediatrics, Université de Montréal, Montreal, QC, Canada; ^4^Endocrinology Service and Research Centre, CHU Sainte-Justine, Montreal, QC, Canada

**Keywords:** Turner syndrome, psychosocial functioning, growth hormone, self-concept, behavior problems

## Abstract

Despite the long-held belief that growth hormone supplementation provides psychosocial benefits to patients with Turner syndrome (TS), this assumption has never been rigorously tested in a randomized control trial. As a sub-study of the Canadian growth-hormone trial, parent-, and patient-completed standardized questionnaires were used to compare 70 girls with TS who received injections (GH group) and 61 similarly followed untreated TS controls (C) on multiple facets of psychosocial functioning. Questionnaires were given (i) at baseline (session 1, mean age = 10.4 y), (ii) before estrogen therapy for puberty induction (session 2, mean age = 13.0 y), (iii) after 1 year of estrogen therapy (session 3, mean age = 14.4 y), and (iv) when growth stopped (session 4, mean age = 16.3 y). Groups were compared for multiple facets of psychosocial function within social, behavioral, self-esteem, and academic domains. Results were also correlated with indices of adult height. We found no global (i.e., across-session) group differences on any scales or subscales of the four domains. In both GH and C groups, age-related improvements were seen for social problems, externalizing behavior problems, and school functioning and age-related declines for social competence and social relations. Both parents and patients claimed GH received less teasing than C but C had more friends than GH. Results from analyses conducted within individual sessions showed that while GH at early sessions claimed to be more popular, more socially engaged, better adapted, and to have higher self-esteem than C, C was reported to be less anxious, depressed, and withdrawn than GH at adult height. The correlation analyses revealed different effects of adult height and height gain on outcome for the two groups. In GH, both height parameters were correlated with multiple parent- and/or self-reported indices from the four psychosocial domains, whereas in C, only adult height and two indices (viz., total self-concept and school functioning), were correlated. The observed modest gains in psychosocial functioning for patients with TS treated with GH highlight the need for alternative approaches to assist them in coping with the challenges of their condition.

## Introduction

Shortness relative to genetic height potential is a universal characteristic of Turner syndrome (TS). Due to the relative resistance of the growth plates to growth hormone (GH) action in patients with TS, they typically averaged ~20 cm below their target adult height (1). Consequently, when GH was being extracted in limited quantities from the pituitaries of human cadavers, their growth acceleration was negligible and, so, they were not considered eligible for this treatment (2). With the advent of biosynthetic GH, however, they could now receive supra-physiological doses of GH. Thus, they were expected to show pronounced growth acceleration and increased adult height. Also underlying this expectation was the assumption that their faster growth and taller adult height from this therapy would lead to improved psychosocial adaptation (3).

To date, only two randomized controlled trials (RCT) to adult height have been published on patients with TS, one in Canada comparing GH injections with no injections (4) and one in the U.S. comparing GH and placebo injections (5). Both trials, which followed patients closely until they reached adult height, reported treatment-related height gains of 7.2 and 5.0 cm, respectively. In the only psychosocial report on these patients to date, GH therapy was not seen to affect health-related quality of life (HRQoL) in a small subset of patients from the Canadian RCT tested at age 20 (6). However, the full extent of psychosocial benefits for the larger sample of patients during GH treatment or on reaching adult height is not known.

Described presently are the findings from the majority of participants in the Canadian RCT, who also took part in a concurrent longitudinal study of their psychosocial functioning during the GH trial. Within this sub-study, GH-treated and untreated control (C) patients, and their parents, completed standardized questionnaires at four set intervals from initial randomization until growth cessation. Groups were compared on a large number of endpoints representing four key domains of psychosocial function, namely social, behavioral, self-esteem, and academic characteristics. In addition, correlations were performed between height indices and outcome at trial completion. To our knowledge, comparable data have not been published in the US trial or elsewhere. Therefore, despite data completion for this sub-study more than a decade ago, it still remains the only one to continuously compare multiple facets of psychosocial functioning in GH-supplemented patients *and untreated TS control patients* through to adult height. As such, we believe our findings are unique and still relevant.

## Materials and Methods

### Design and Procedures

Between February 1989 and May 1994, the main study enrolled 154 patients with TS ranging from 7 to 13 years of age. All were prepubertal at study entry. Eligibility criteria for this study were: (a) height below the 10th percentile for chronological age and (b) an annualized height velocity of < 6.0 cm/y [see (4) for additional eligibility criteria and exclusion criteria]. Initially, the patients were stratified into three subgroups based on height relative to chronological age and, then within subgroups, randomized to either a GH-treatment or a no-GH control condition. Treatment involved recombinant human GH (Humatrope Eli Lilly Canada Inc., Toronto, Canada) by subcutaneous injection (dose = 0.3 mg/kg/week) six times weekly. Injections were continued for ~6 years until an annualized height velocity of < 2 cm/y and bone age of 14 y or greater were attained. In addition, all patients with primary ovarian failure (the majority of cases) were given standardized oral estradiol therapy at ~age 13 y; the handful of patients near to or above age 13 at study entry, received this 1 year after commencing GH treatment. The protocol for estradiol therapy involved 0.0025 mg oral ethinyl estradiol daily for the first year, 0.005 mg the next year, and cyclic estrogen and progesterone replacement thereafter.

In the sub-study, which took place between February 1989 and December 2002, nurse practitioners from 13 pediatric endocrine clinics across Canada (7) gave families packets of questionnaires printed in English or French. These were provided at four preset intervals: initiation of the trial or “session 1”; just prior to estrogen therapy or “session 2”; after 1 year of estrogen therapy or “session 3”; and when growth stopped or “session 4.” Completed questionnaires were returned via mail to The Hospital for Sick Children (SickKids), where research assistants blinded to treatment status scored the tests, maintained the database, and conducted preliminary data analyses. The final analyses were conducted more recently by JR.

All procedures were carried out in accordance with the guidelines of the ethics review committees of each participating institution (Supplementary Table 1), which provided approval for both the main study and this sub-study. The SickKids Research Ethics Board provided additional approval for sub-study data coordination and analyses at this facility. All parents gave informed consent while patients gave informed assent or consent. The main trial was registered with ClinicalTrials.gov Identifier NC700791113.

### Participants

**Figure 1:**
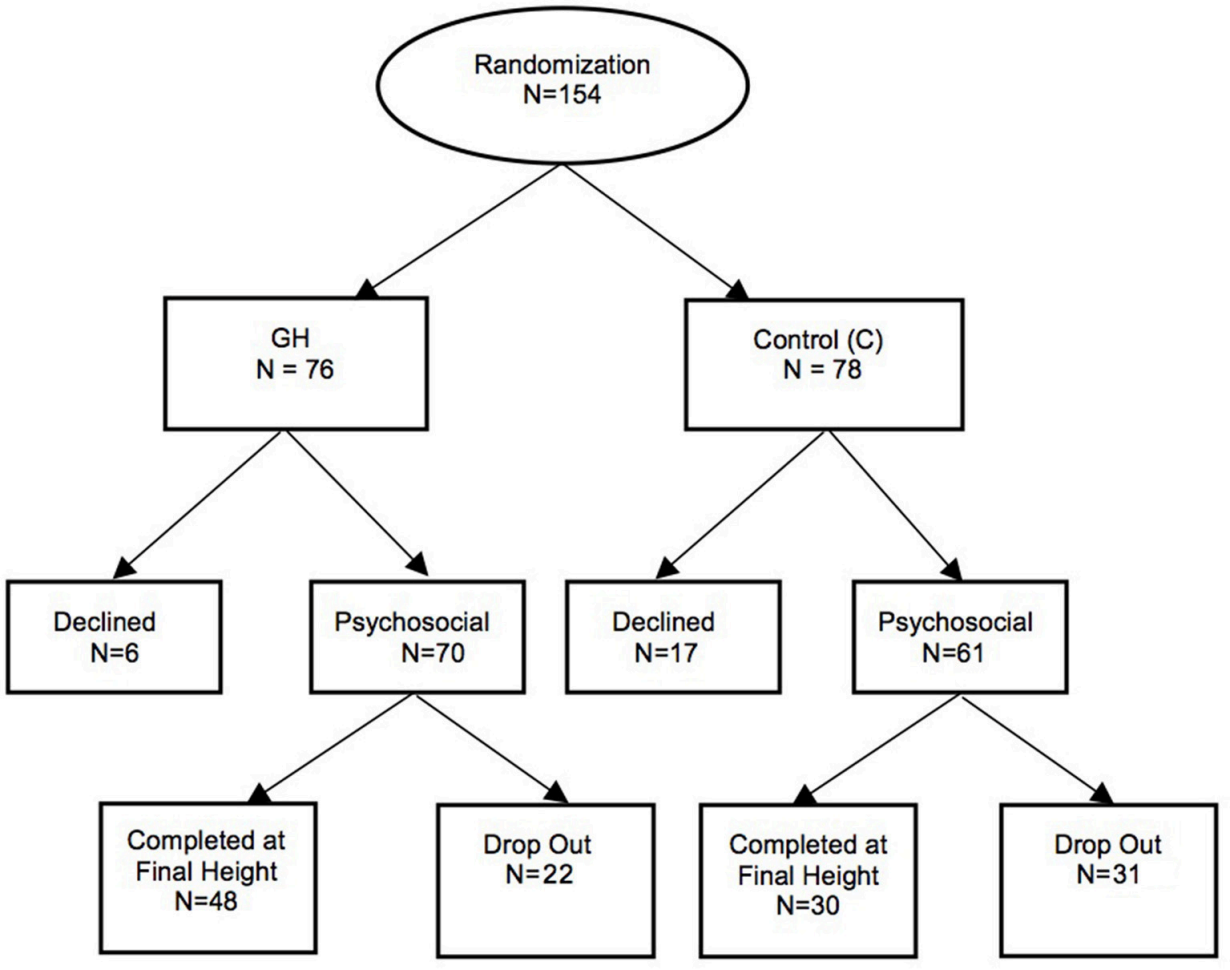
Outline of participation in the psychosocial sub-study.

One hundred and thirty-one of the original 154 patients took part in the sub-study (Figure 1). Primary reason for not joining was unwillingness to be involved. Shown in Supplementary Table 1 are the patient numbers from each participating endocrine clinic. Three children switched sites during the trial due to family relocation.

Of the 131 sub-study participants, 70 belonged to the GH group and 61 to the C group representing 92 and 70% of main-study groups, respectively. One hundred and twenty-two patients (67 GH, 55 C) began at baseline (session 1) while a further nine (3 GH, 6 C), from a site whose PI was initially unwilling to participate, entered at session 2. Sessions 2 and 3 had 111 (67 GH, 44 C) and 82 (53 GH, 29 C) patients, respectively. The differential drop-out for C vs. GH between sessions 2 and 3 may be partially explained by a physician offering treatment independent of the trial to his C patients. Seventy-eight participants (48 GH, 30 C) completed the sub-study at session 4, representing 77 and 70% of GH and C main-study completers and 72 and 54% of participants who began the sub-study, respectively. Completers did not differ from drop-outs in terms of baseline sociodemographic characteristics (data not shown).

### Tests and Measures

Parents initially filled out a brief demographic questionnaire seeking information on marital status and education/occupation used to derive a 5-point index of socioeconomic status (SES) (1 = high status) (8); they also completed the Child Behavior Checklist (CBCL) (9) at sessions 1 to 4. Patients completed the Youth Self-Report (YSR) (10) at sessions 2 to 4 and the Piers-Harris Children's Self-Concept Scale (PHCSCS) (11) at all four sessions.

The CBCL is a widely used standardized questionnaire based on parent report that assesses behavior problems in 4–16-year olds. It contains a series of open-ended questions that are used to derive the following four social-competence (SC) scales: Total Social Competence, Activities, Social Relations, and School. The CBCL also provides a list of 113 factual statements such as “acts too young for age” and “feels worthless or inferior.” Parents use a three-point scale (1 = not true; 2 = somewhat or sometimes true; 3 = very true or often true) to rate their daughters on these items. Computerized scoring of the items yields: a Total Behavior Problems (BP) index and scores for Internalizing and Externalizing Problems broad-band scales and the eight narrow-band scales of Withdrawn, Somatic-complaints, Anxious/Depressed, Social, Thought, Attention, Delinquency, and Aggression problems. Internalizing and Externalizing Problems scales are derived from a subset of narrow-band subscales that does not include Social, Thought, or Attention Problems. For SC indices, a higher positive score signifies better functioning and for BP, more behavior problems. Although results were originally generated as T-scores (mean = 50; SD = 10) based on normative test data, we converted them to SD units (mean = 0; SD = 1) as per (12). Somatic Complaints results are not reported presently.

In addition, we separately recorded scores from three of the CBCL items, namely “number of friends” and “time with friends” from the SC component and “gets teased a lot” (item #38) from the BP component. The first two were based on a four-point scale (4 = most favorable rating) and the third, a 3-point scale (see above). As part of the School subscale, parents also rated their daughter's reading and math abilities via a 4-point scale (4 = very good) and indicated her grade at school and if she was in a special class, had failed a grade, or had academic problems.

The YSR is a self-report instrument with a similar structure and scoring system as the CBCL but in the version we provided, lacked information on academics. Scores from two individual items were additionally recorded: “I get teased a lot” (#38) and “I feel lonely” (#12). Because the YSR is only first administered at age 11, this questionnaire was not provided until session 2.

The PHCSCS is a self-report questionnaire consisting of 80 statements such as “I am a good person” or “I am popular with boys.” Patients indicated if statements were true or false of themselves. Scoring yielded a Total Self Concept index plus scores for six subscales: Behavioral Adjustment, Intellectual & School Status, Physical Appearance, Freedom from Anxiety, Popularity, and Happiness/Satisfaction. All PHCSCS results were reported as percentiles based on test norms with higher scores signifying more favorable self-esteem.

For present purposes, results from the three questionnaires were examined within four domains of psychosocial functioning, namely social abilities, behavior problems, self-esteem, and academics. Each domain was derived from the relevant subscales or items of the various questionnaires. Social functioning was based on (a) CBCL and YSR Total Social Competence, Activities, Social Relations, and Social Problems scales, (b) the PHCSCS Popularity index, and (c) selected CBCL and YSR items. Likewise, the behavior-problem domain was represented by (a) CBCL and YSR Total and Internalizing and Externalizing Problems scores, (b) selected CBCL and YSR narrow-band scores (viz., Withdrawn, Anxious/Depressed, Thought Problems, Attention Problems, Delinquency, and Aggression), and (c) Behavioral Adjustment and Freedom from Anxiety scores from the PHCSCS. Self-esteem was based on the four remaining PHCSCS scales, namely Total Self Concept, Intelligence/School, Physical Appearance, and Happiness/Satisfaction. Academic functioning was based on the CBCL School scale and Reading and Math scores.

### Data Analysis and Statistics

Groups were compared at baseline and subsequent sessions using an intent-to-treat analysis. Missing baseline data from the nine patients first entering the psychosocial study at session 2 were imputed using the mean scores of the child's height-for-age stratification subgroup; note, these patients did provide baseline height data. For the other missing data from session 2, scores were imputed using a next-observation-carried-backward approach and for missing data from sessions 3 and 4, a last-observation-carried-forward approach based on (13). If a subject had data from sessions 2 and 4 but not session 3, the mean of her session 2 and 4 scores was used.

All data were analyzed using SPSSv24 (14). *t*- and χ^2^ tests served to compare groups for demographics and height. For CBCL and PHCSCS questionnaires, post-baseline data were analyzed using mixed-model repeated-measures analyses of covariance (ANCOVA) with Group as the between-subjects factor, Session as the repeated factor, and baseline (i.e., session 1) scores as the covariate. Since the YSR was not administered until session 2 (see above), results for this questionnaire were analyzed by repeated-measures analyses of variance (ANOVA) with Group as the between-subjects factor and Session as the repeated measure. In order to identify whether groups also differed at individual sessions, multivariate analyses of variance (MANOVA) were performed within sessions for all measures belonging to a domain. A power analysis indicated that with 70 and 61 participants per group (average = 65) and with an α of 0.05 and β of 80%, we could detect moderate effect sizes (d = ~0.45) (15).

To evaluate the impact of adult height and height gain on psychosocial indices, we performed for GH and C groups separately, two series of Pearson correlations between adult height indices and measures of outcome at the final session. Separate series of correlations were conducted for each of the four psychosocial domains. For these analyses, only data from sub-study completers (i.e., no imputed values) were used.

For between-group comparisons, the *p*-value was set at 0.05 using a two-tailed test with the Bonferonni *p*-correction applied if a test had multiple subscales within a domain (e.g., *p*-values for two narrow-band Externalizing subscales were divided by 2). A similar correction approach was applied to the correlations, but a one-tailed test was instead used, given the assumption of better outcome following greater growth (3).

## Results

### Demographics

For the combined sample, ages reported as mean ± SD at the four sessions were: 10.4 ± 1.6 y at session 1; 13.0 ± 1.0 y at session 2; 14.4 ± 0.6 y at session 3; and 16.3 ± 1.0 y at session 4. Corresponding school grades were 4.7, 7.4, 8.8, and 10.5, respectively. Table 1 presenting the groups' baseline characteristics shows GH and C did not differ in age, grade, SES, percent English speaking, grade failure, special education, academic problems or experiencing divorce, separation, or death.

**Table 1:** Demographic characteristics at baseline.

	**GH1^**a**^**	**Control^**a**^**
Age in years^b^	10.3 (1.7)	10.5 (1.5)
Grade at School^b^	4.6 (1.7)	4.8 (1.4)
SES Class^b^	2.6 (0.9)	2.5 (0.8)
% English Speaking	70.6	75.4
% Failed a grade	20.0	19.3
% In special class	17.5	14.0
% Academic problems	38.3	33.3
% Experiencing divorce etc.	13.6	23.2

a Baseline sample based on 64 GCH and 50 C cases at session 1;

b *Results are expressed as mean (SD)*.

### Height Data

Table 2 presents the height data of the sub-study participants. Target height, while included in the 2005 paper (4), was not used in current analyses of height response or correlations with outcome data.

**Table 2:** Mean (SD) height data^a^.

	**Session 1**		**Session 2**		**Session 3**		**Session 4**	
	**GH**	**C**	**GH**	**C**	**GH**	**C**	**GH**	**C**
Height (cm)	119.5 (18.5)	120.1 (8.3)	**127.0 (8.4)**	**123.9 (8.4)**	**138.3 (7.1)**	**130.7 (6.0)**	**147.1 (6.4)**	**136.5 (9.2)**
SD (TS norms)	−0.10 (0.9)	−0.17 (0.8)	**0.59 (1.0)**	**−0.15 (0.9)**	**1.32 (1.2)**	**0.60 (1.0)**	**0.60 (0.9)**	**−0.10 (0.9)**
SD (NCHS)	−3.21 (0.8)	−3.28 (0.8)	**−2.80 (1.0)**	**−3.49 (0.8)**	**−2.80 (1.0)**	**−3.89 (0.8)**	**−2.26 (0.9)**	**−3.52 (0.9)**

a *Data shown in bold indicate significant group differences*.

Current results are similar to those for the full sample in the main study (4). Groups were both very short initially (mean baseline height below −3 SD) and similar to each other. Subsequently, however, GH was taller than C (*p* < 0.05 session 2; *p* < 0.001 sessions 3 and 4) as well as differed significantly in height gain (*p* < 0.001). GH, between sessions 1 and 4, gained 27.7 ± 9.4 cm, representing a unit change on the NCHS scale of 1.0 SD, whereas C gained only 16.2 ± 7.3 cm representing a unit change of −0.2 SD. For both groups combined, a greater height gain was correlated with a younger age at study entry (*p* < 0.001) while for GH only, taller adult height and younger age at study entry were also correlated (*p* < 0.01).

Since all height indices were significantly correlated (*p* < 0.001) within and across sessions, we chose two height parameters for further analyses in order to reduce the number of correlations with outcome: adult height in NCHS SD units and height gain (cm) between sessions 1 and 4.

### Social Functioning

Table 3 presents the post-baseline scores for all social functioning indices while Supplementary Tables 2 and 3 contain the baseline scores from CBCL and PHCSCS questionnaires, respectively. Table 3 also shows the statistical results from MANCOVAs on CBCL and PHCSCS tests and MANOVA on the YSR. Table 4 provides the findings from the correlational analyses computed between height and outcome indices.

**Table 3:** Mean (SE) scores on social functioning indices at post-baseline sessions^a^.

	**Session 2**		**Session 3**		**Session 4**		**F-values**^**b**^		
	**GH**	**C**	**GH**	**C**	**GH**	**C**	**GP**	**Session**	**GPXSession**
**CBCL**									
Total Social Competence^c^	−0.77 (0.10)	−0.83 (0.10)	−0.81 (0.10)	0.74 (0.08)	−0.93 (0.09)	−1.00 (0.07)	0.18	5.63^**^	1.02
Activities^c^	−0.37 (0.08)	−0.57 (0.08)	−0.43 (0.09)	0.45 (0.08)	−0.55 (0.09)	−0.75 (0.08)	1.10	0.68	2.13
Social Relations^c^	−0.69 (0.09)	−0.82 (0.09)	−0.81 (0.10)	0.73 (0.09)	−0.92 (0.09)	−0.89 (0.08)	0.35	5.23^**^	0.42
Social Problems^d^	1.07 (0.08)	1.15 (0.10)	0.98 (1.00)	0.86 (0.09)	0.93 (1.00)	0.70 (0.06)	0.53	20.46^***^	2.58^+^
Number of friends^e^	2.88 (0.10)	3.03 (0.11)	2.96 (0.09)	3.06 (0.09)	**2.99 (0.08)**	**3.20 (0.07)**	2.50^+^	20.30^***^	0.80
Time with friends^e^	2.08 (0.10)	2.05 (0.10)	2.00 (0.09)	2.03 (0.08)	1.93 (0.07)	2.02 (0.06)	0.08	2.41	0.47
Teased^f^	0.70 (0.10)	0.93 (0.10)	0.69 (0.08)	0.80 (0.08)	0.66 (0.07)	0.50 (0.07)	0.13	0.23	5.27^**^
**YSR**									
Total Social Competence^c^	−0.60 (0.10)	−0.82 (0.09)	–0.63 (0.11)	0.45 (0.11)	−0.78 (0.12)	−0.72 (0.10)	0.11	2.18	2.59^+^
Activities^c^	***–0.22 (0.09)***	***–0.62 (0.08)***	−0.44 (0.09)	0.46 (0.09)	−0.49 (0.10)	−0.64 (0.09)	3.37^+^	2.87^+^	3.38^*^
Social Relations^c^	−0.58 (0.08)	−0.75 (0.08)	**–0.70 (0.10)**	**0.37 (0.11)**	−0.82 (0.10)	−0.66 (0.08)	1.08	3.82^*^	5.65^**^
Social Problems^d^	0.48 (0.12)	0.51 (0.07)	0.48 (0.09)	0.55 (0.07)	*0.52 (0.10)*	*0.77 (0.09)*	1.83	1.46	0.95
Teased^f^	0.64 (0.10)	0.78 (0.07)	***0.48 (0.09)***	***0.93 (0.09)***	*0.49 (0.09)*	*0.72 (0.09)*	8.05^**^	1.66	3.39^*^
Lonely^f^	0.32 (0.07)	0.42 (0.07)	0.34 (0.08)	0.37 (0.10)	0.42 (0.07)	0.55 (0.07)	1.47	1.88	0.47
**PHCSCS**									
Popularity^=^g^^	***57.4 (3.8)***	***42.6 (4.0)***	*57.7 (3.4)*	*48.3 (3.7)*	53.6 (3.4)	54.2 (2.6)	20.74^+^	17.33^***^	30.23*

a See Supplementary Tables 2 and 3 for CBCL and PHCSCS Session 1 results;

b MANCOVA for CBCL and PHCSCS with Session 1 results as covariate; MANOVA for YSR;

c Expressed in SD units with negative scores indicating suboptimal social functioning;

d Expressed in SD units with positive scores signifying more problems;

e Scored on a 4-point scale (1 = “none”; 2 = “1”; 3 = “2 or 3”; 4 = “4 or more”);

f Scored on a 3-point scale (1 = “not true”; 2 = “somewhat or sometimes true”; 3 = “very true or often true”);

g Expressed in percentile scores; Results in bold italics indicate significant group difference at p < 0.01 level corrected; results in bold indicate significant group difference at p < 0.05 level corrected; results in italics indicate a trend-level difference at p < 0.10 level;

*** p < 0.001;

** p < 0.01;

* *p < 0.05; +indicates a trend-level difference at p < 0.10 level*.

**Table 4:** Correlations between height parameters and social functioning indices at session 4^a^.

	**Adult Height (NCHS SD Units)**		**Δ** **Height**	
	**GH**	**C**	**GH**	**C**
**CBCL**				
Total Social Competence	0.394^**^	0.201	0.252^*^	0.007
Activities	0.209	0.007	0.161	−0.179
Social Relations	0.363^**^	0.121	0.252^*^	0.047
Social Problems	−0.285^*^	−0.074	−0.140	−0.147
Number of friends	0.041	0.127	0.052	−0.070
Time with friends	0.239^*^	0.223	0.088	−0.084
Teased	−0.251^*^	−0.154	−0.098	−0.016
**YSR**				
Total Social Competence	0.329^**^	0.257	0.225^*^	0.104
Activities	0.379^**^	0.003	0.168	0.004
Social Relations	0.368^**^	0.254	0.187	0.111
Social Problems	0.018	−0.128	−0.087	−0.216
Teased	−0.065	−0.178	−0.174	−0.061
Lonely	−0.181	−0.152	−0.248^*^	−0.054
**PHCSCS**				
Popularity	0.143	0.129	0.271^*^	0.083

a Results are based on a one-tailed test;

** p < 0.01,

* *p < 0.05*.

According to parents, GH and C both scored quite poorly on CBCL Total Social Competence (range = 0.7–1.0 SD units below population norms) and Social Problems indices (~1.0 SD units above population norms). Although significant Group effects were not seen for any of these indices, significant Session effects were observed reflecting age-related declines in Total Social Competence (*p* < 0.01) and Social Relations (*p* < 0.01) and an age-related improvement Social Problems (*p* < 0.001). Also, for Social Problems, a trend-level Group X Session interaction (*p* < 0.10) reflected the somewhat greater improvement over time by C than GH. There were no group differences for any of the baseline social-functioning indices.

**Figure 2:**
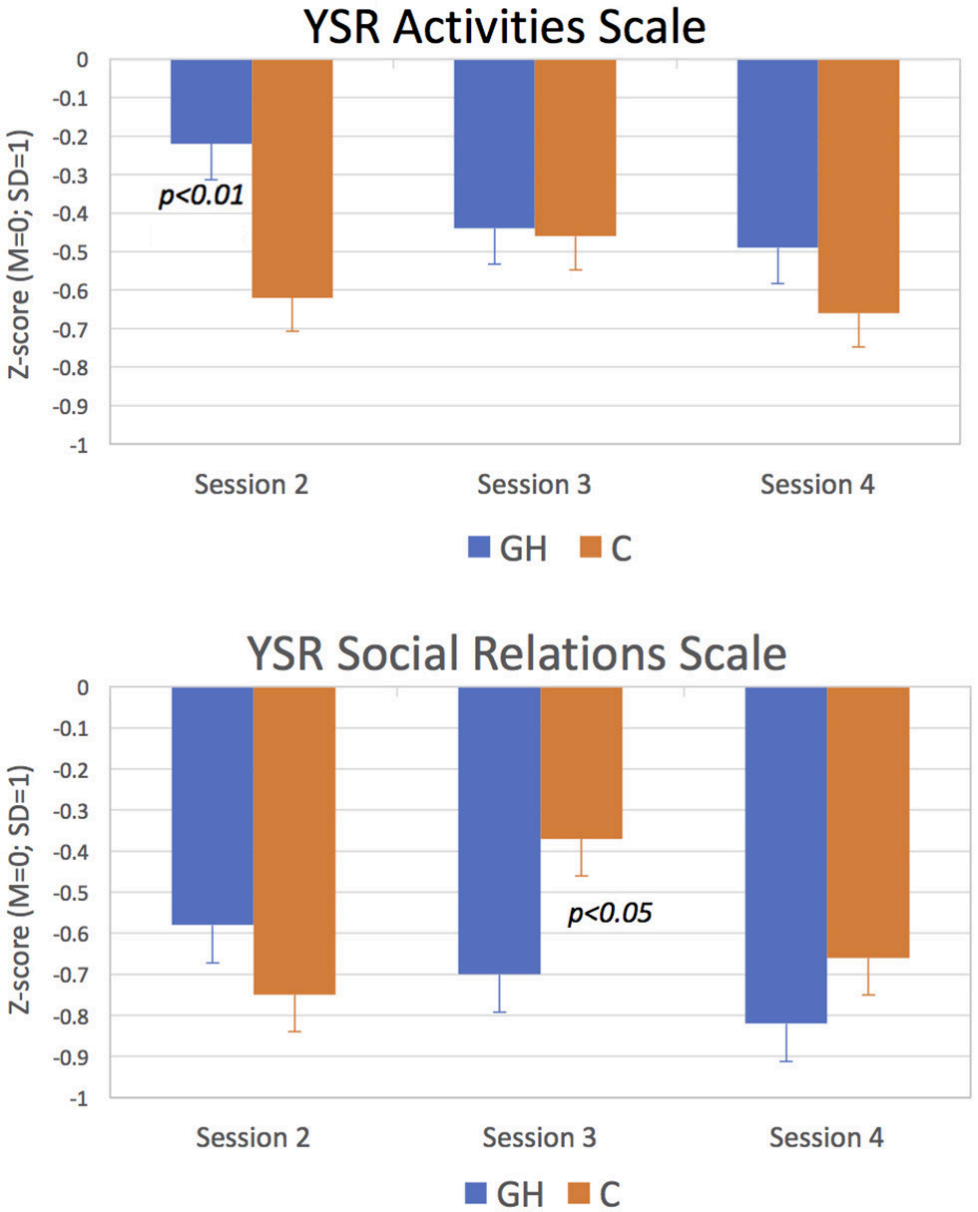
Scores for YSR Activities and Social Relations Scale. GH scored significantly above C on Activities at Session 2 and significantly below C on Social Relations at Session 3 (*p* < 0.05 for both).

**Figure 3:**
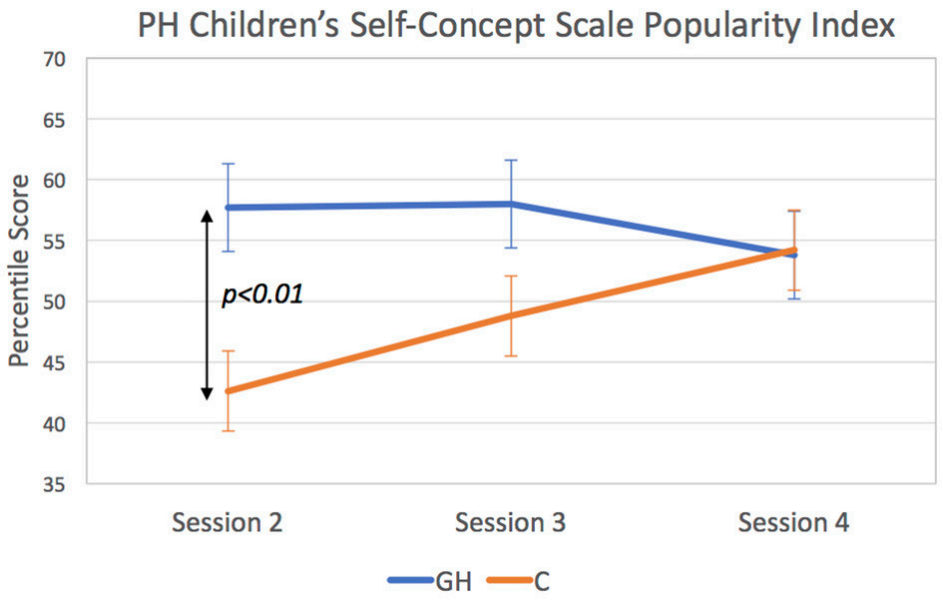
Scores for Piers Harris Children's Self-Concept Scale Popularity Index. GH scored significantly above C at Session 2 (*p* < 0.01).

For the patient-completed YSR and PHCSCS questionnaires, results indicated no omnibus (i.e., across-session) Group effects on any of the scales or subscales. However, groups showed different patterns of change as indicated by significant Group X Session interactions for YSR Activities (*p* < 0.05), YSR Social Relations (*p* < 0.01), and PHCSCS Popularity (*p* < 0.05), and a trend-level interaction (*p* < 0.10) for YSR Total Social Competence. Figure 2 depicting the results for YSR Activities and Social Relations scales and Figure 3, for PHCSCS Popularity, show that GH initially outscored C but that C later improved scoring comparably to or beyond GH at adult height. Results from analyses based on within-session results indicated GH at session 2 claimed more social engagement and popularity than C (*p* < 0.01), whereas parents reported C had better social relations (*p* < 0.05) at session 3. Groups did not differ in their baseline results.

**Figure 4:**
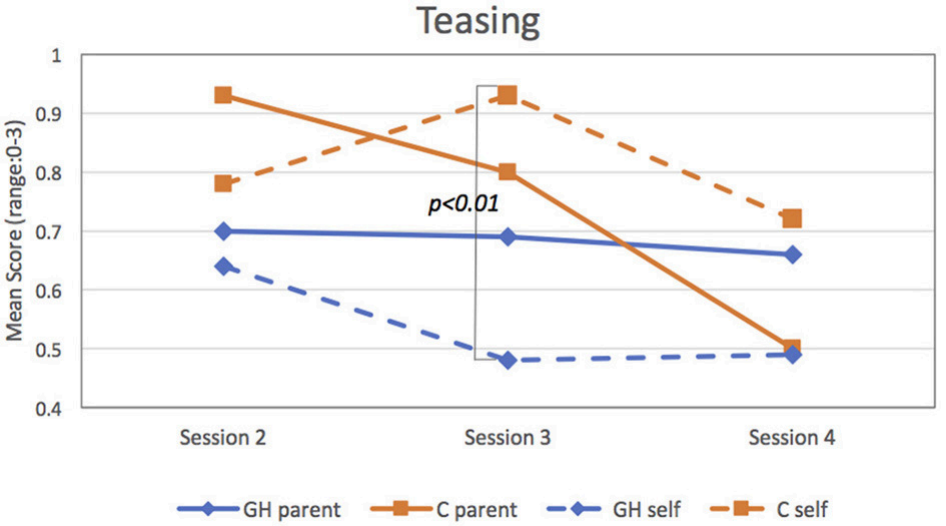
Scores for parents (solid lines) and patients (hatched lines) on “Teasing” item. Patients reported significantly less teasing for GH than C at Session 3 (*p* < 0.01).

For the individual items, findings revealed C had overall more friends than GH (*p* < 0.10), especially at session 4 (*p* < 0.05), but groups did not differ in time spent with friends or reported loneliness. Regarding being teased, both parents and patients reported high teasing rates for all patients, and these exceeded the rates for patients with psychological problems in the normative reference sample (16). According to parents (CBCL), the Group X Session interaction for teasing was significant (*p* < 0.01) while on self-report (YSR), both Group (*p* < 0.01) and the Group X Session interaction (*p* < 0.05) were significant. Figure 4 depicting these findings shows that parents (solid lines) claimed GH experienced a constant level of teasing across sessions, whereas C was teased initially more than GH but by session 4, teased less. According to patients (hatched lines), GH experienced consistently less teasing than C, especially at session 3 (*p* < 0.01).

The correlational findings indicated height influenced parent- and/or patient-reported social functioning, but only in GH. For these patients, taller adult height was correlated with higher levels of social competence and better social relations (*p* < 0.01 for both), more social engagement (*p* < 0.01), fewer social problems (*p* < 0.05), more time with friends (*p* < 0.05), and less teasing (*p* < 0.05) while a larger height gain was associated with better self-esteem (*p* < 0.05) and social relations (*p* < 0.05), greater popularity (*p* < 0.05), and less loneliness (*p* < 0.05). There were no significant correlations for C.

### Behavior Problems

Table 5 presents the post-baseline BP results while Supplementary Tables 2 and 3 contain the baseline scores for CBCL and PHCSCS tests, respectively (YSR not administered at session 1). Table 6 presents the correlational findings.

**Table 5:** Mean Post-Baseline (SE) Scores on Behavior Problem Indices^a^.

	**Session 2**		**Session 3**		**Session 4**		**F-values**^**b**^		
	**GH**	**C**	**GH**	**C**	**GH**	**C**	**GP**	**Session**	**GP X Session**
**CBCL**^**c**^									
Total problems	0.62 (0.07)	0.66 (0.07)	0.49 (0.05)	0.49 (0.05)	0.46 (0.05)	0.38 (0.04)	0.58	20.04^***^	1.29
Internalizing problems	0.53 (0.06)	0.57 (0.06)	0.42 (0.05)	0.46 (0.05)	0.42 (0.05)	0.34 (0.04)	0.15	8.18^***^	2.14
Externalizing problems	0.43 (0.07)	0.44 (0.08)	0.28 (0.04)	0.32 (0.05)	0.27 (0.05)	0.23 (0.03)	0.21	35.90^***^	0.67
Withdrawn	0.56 (0.08)	0.56 (0.07)	0.45 (0.06)	0.42 (0.07)	***0.54 (0.06)***	***0.33 (0.07)***	1.00	11.78^***^	3.73^*^
Anxious/depressed	0.49 (0.08)	0.60 (0.08)	0.41 (0.04)	0.47 (0.04)	**0.47 (0.07)**	**0.27 (0.07)**	1.03	6.50^**^	3.62^*^
Thought problems	0.56 (0.08)	0.54 (0.09)	0.43 (0.06)	0.43 (0.07)	0.31 (0.05)	0.34 (0.05)	0.18	4.59^**^	0.29
Attention problems	0.89 (0.11)	0.93 (0.11)	0.67 (0.08)	0.59 (0.07)	0.50 (0.07)	0.51 (0.05)	0.28	23.89^***^	0.64
Delinquency	0.37 (0.07)	0.41 (0.08)	0.22 (0.05)	0.24 (0.05)	0.24 (0.05)	0.18 (0.03)	0.05	15.43^***^	0.94
Aggression	0.48 (0.08)	0.46 (0.07)	0.34 (0.05)	0.40 (0.07)	0.31 (0.06)	0.27 (0.04)	0.24	30.41^***^	1.43
**YSR**^**c**^									
Total problems	0.22 (0.08)	0.26 (0.03)	0.49 (0.05)	0.48 (0.05)	0.27 (0.05)	0.29 (0.03)	0.13	16.55^***^	0.07
Internalizing problems	0.23 (0.08)	0.29 (0.03)	0.20 (0.03)	0.24 (0.03)	0.29 (0.05)	0.28 (0.04)	0.34	2.80	0.38
Externalizing problems	0.18 (0.08)	0.15 (0.03)	0.28 (0.05)	0.32 (0.05)	0.14 (0.06)	0.13 (0.02)	0.00	8.03^***^	0.31
Withdrawn	0.24 (0.10)	0.23 (0.05)	0.29 (0.07)	0.28 (0.07)	*0.39 (0.07)*	*0.34 (0.06)*	0.23	2.85	0.26
Anxious/depressed	0.12 (0.08)	0.21 (0.04)	0.23 (0.05)	0.25 (0.06)	0.29 (0.06)	0.26 (0.05)	0.16	1.36	0.80
Thought problems	0.12 (0.09)	0.26 (0.05)	0.12 (0.03)	0.14 (0.04)	0.14 (0.05)	0.13 (0.02)	0.98	0.72	0.78
Attention problems	0.22 (0.09)	0.27 (0.06)	0.24 (0.05)	0.21 (0.05)	0.34 (0.07)	0.27 (0.04)	0.13	2.21	0.55
Delinquency	0.12 (0.09)	0.11 (0.02)	0.16 (0.04)	0.12 (0.04)	0.15 (0.05)	0.11 (0.02)	0.46	0.24	0.33
Aggression	0.23 (0.09)	0.20 (0.04)	0.17 (0.04)	0.15 (0.04)	0.20 (0.06)	0.15 (0.03)	0.44	0.61	0.10
**PHCSCS**^**d**^									
Behavioral adaptation	78.5 (2.7)	71.8 (2.9)	**83.9 (2.0)**	**75.4 (2.7)**	80.4 (2.1)	75.1 (2.4)	2.42	13.53^***^	0.74
Freedom from anxiety	63.2 (3.3)	57.7 (3.6)	67.9 (3.1)	60.7 (3.2)	58.1 (3.8)	58.9 (2.8)	0.74	11.10^***^	2.32

a See Supplementary Tables 1, 2 for Session 1 results;

b MANCOVA for CBCL and PHCSCS with Session 1 results as covariate; MANOVA for YSR;

c Expressed in SD units with higher positive scores reflecting more behavior problems and negative scores indicating very good behavior;

d Expressed in percentile scores NOTE: Results shown in italics indicate significant group difference at p < 0.05 level corrected and in bold italics at p < 0.01 level corrected;

*** p < 0.001;

** p < 0.01;

* *p < 0.05; ^**+**^indicates a trend at the p < 0.10 level*.

**Table 6:** Correlations between height parameters and behavior problem results at session 4.

	**Adult height (NCHS SD Units)**		**Δ** **Height**	
	**GH**	**C**	**GH**	**C**
**CBCL**				
Total problems	−0.166^a^	−0.184	−0.184	0.100
Internalizing problems	−0.081	−0.146	−0.195	0.087
Externalizing problems	−0.095	−0.129	−0.090	0.205
Withdrawn	−0.067	0.006	−0.181	−0.182
Anxious/depressed	−0.033	0.077	−0.157	−0.079
Thought problems	−0.128	−0.151	−0.164	−0.209
Attention problems	−0.172	−0.010	−0.153	−0.047
Delinquency	−0.010	0.004	−0.067	0.071
Aggression	−0.142	−0.042	−0.091	−0.017
**YSR**				
Total problems	−0.132	−0.116	−0.190	−0.089
Internalizing problems	−0.067	−0.012	−0.187	−0.110
Externalizing problems	−0.025	−0.214	−0.185	−0.074
Withdrawn	−0.068	−0.149	−0.250^*^	0.050
Anxious/depressed	−0.075	−0.096	−0.257^*^	−0.172
Thought problems	−0.137	0.167	−0.131	−0.145
Attention problems	−0.050	−0.046	−0.192	0.008
Delinquency	0.096	0.014	−0.131	−0.116
Aggression	0.125	−0.053	−0.165	−0.112
**PHCSCS**				
Behavioral adaptation	0.099	0.227	0.164	0.028
Freedom from anxiety	0.232	0.260	0.347^**^	0.053

a All results are based on a one-tailed test;

*** p < 0.01;

* *p < 0.05*.

For all CBCL and YSR BP indices, no omnibus (i.e., across-session) Group effects were observed. However, most scales indicated significant Session effects reflecting age-related reductions within both groups, particularly between sessions 2 and 3. Significant Group X Session interactions for Withdrawn and Anxious/depressed CBCL scales (*p* < 0.05 for both) indicated that the groups manifested different patterns of change over time. Specifically, C showed greater improvement than GH on Withdrawn Problems, as well as a steady improvement on the Anxious/depressed scale. GH, by contrast showed little change across sessions. At session 4, C scored significantly below GH on Withdrawn (*p* < 0.01) and Anxious/depressed (*p* < 0.05) problem scales.

For the two PHCSCS BP scales of Behavioral Adaptation and Freedom from Anxiety, findings revealed only significant Session effects (*p* < 0.001 for both indices). For Behavioral Adaptation, results reflected a steady improvement with time; also, at session 3, GH outscored C (*p* < 0.05). Results indicated a steady decline on Freedom from Anxiety for both groups, suggesting increased anxiety with age.

**Figure 5:**
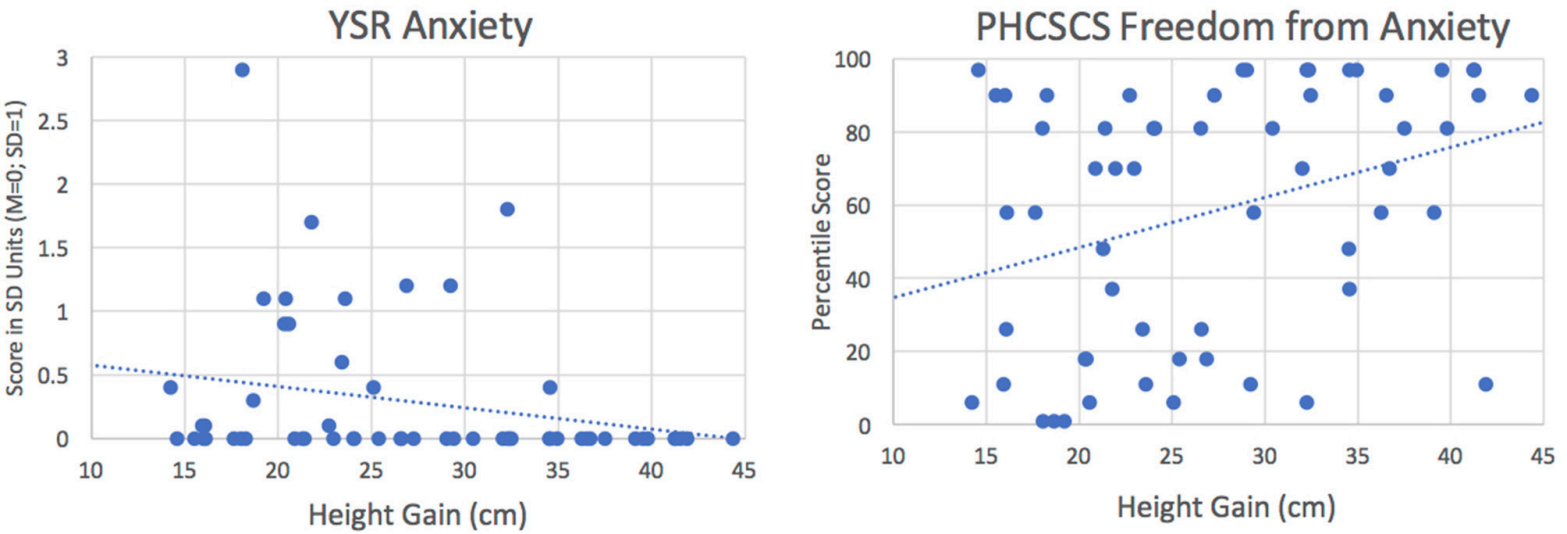
Correlations between height gain and anxiety in GH group. Left: Correlation with YSR Anxiety, *y* = 0.75–0.017*x, r* = −0.257, *p* < 0.05; Right: Correlation with PHCSCS Freedom from Anxiety, *y* = 21.1+1.37*x, r* = 0.347, *p* < 0.001. Results indicate that children showing larger height gains have less anxiety.

Table 6 shows fewer significant associations for BP than for SC scales (section Social Functioning) and only within the GH group, for whom a larger height gain was associated with lower (i.e., better) YSR Withdrawn and Anxious/depressed scores and higher PHCSCS Freedom from Anxiety scores (Figure 5).

### Self-Esteem

Table 7 presents the post-baseline results for the four PHCSCS indices evaluating self-esteem, namely Total Self-Concept, Intelligence, Physical Appearance, Happiness/Satisfaction. Supplementary Table 3 contains the baseline scores for these indices and Table 8, the correlation results.

**Table 7:** Mean (SE) post-baseline piers-harris children's self-concept scale and academic scores.

	**Session 2**		**Session 3**		**Session 4**		**Group X Session**		
	**GH**	**C**	**GH**	**C**	**GH**	**C**	**Group**		**Session**
**PHCSCS**									
Total self concept^a^	71.0 (3.04)	68.6 (2.7)	**78.2 (2.6)**	**69.1 (2.9)**	72.3 (2.9)	71.0 (2.5)	1.10	9.40^***^	3.58^*^
Intellectual and school^a^	68.8 (3.1)	70.3 (3.3)	72.4 (2.7)	70.9 (2.9)	66.3 (3.0)	72.1 (24)	0.14	4.74^**^	1.96
Physical appearance^a^	55.0 (3.0)	62.5 (3.2)	58.4 (3.1)	54.6 (3.0)	60.0 (2.2)	59.6 (2.2)	0.02	11.09^***^	2.91^+^
Happiness and satisfaction^a^	66.5 (2.8)	70.2 (3.0)	74.2 (2.8)	66.4 (3.1)	70.0 (2.9)	68.2 (2.7)	1.69	9.40^***^	2.27
**CBCL**									
School^b^	−0.80 (0.07)	−0.70 (0.07)	−0.66 (0.09)	−0.76 (0.091)	−0.44 (0.08)	−0.72 (0.08)	0.93	15.62^***^	1.43
Reading^c^	3.27 (0.07)	3.35 (0.07)	3.36 (0.07)	3.35 (0.07)	3.27 (0.07)	3.27 (0.07)	0.07	11.95^***^	0.39
Math^c^	2.73 (0.08)	2.86 (0.08)	2.79 (0.08)	2.89 (0.08)	2.85 (0.08)	2.83 (0.08)	0.72	3.06^*^	0.81

a Results are expressed as percentile scores;

b Expressed as z-scores based on normative sample for tests (mean = 0; SD = 1), positive score signifies better outcome;

c Based on a 4-point scale (4 = very good); Results shown in bold indicate significant group difference at the p < 0.05 level;

*** p < 0.001;

** p < 0.01;

* *p < 0.05*.

**Table 8:** Correlations between Height Parameters and Self-Esteem and Academic Functioning Indices at Session 4^a^.

	**Final Height (NCHS SD Units)**		**Δ** **Height**	
	**GH**	**C**	**GH**	**C**
**SELF-ESTEEM**				
Total self-concept	0.208	0.289^*^	0.362^**^	−0.037
Intelligence & school^b^	0.227	0.176	0.384^**^	0.045
Physical appearance^b^	0.200	0.172	0.323^**^	0.071
Happiness/satisfaction^b^	0.214	0.164	0.374^**^	−0.022
**ACADEMIC/COGNITIVE FUNCTIONING**				
CBCL school scale	0.276^*^	0.316^*^	0.082	0.148
Reading rating^b^	0.191	−0.104	0.100	0.065
Math rating^b^	0.099	0.029	0.102	0.040

a Results are based on a one-tailed test;

b Critical p-value divided by 3 for three indices in the category;

** p < 0.01;

* *p < 0.05 after correction for 3 multiple scales*.

No omnibus (i.e., across session) Group effects were observed for any of the self-esteem indices. However, a significant Group X Session interaction for Total Self Concept (*p* < 0.05) reflected the increasingly better self-esteem among GH than C. GH also scored significantly above C at session 3 (*p* < 0.05) in Total Self Concept. A trend-level interaction for Physical Appearance (*p* < 0.10) reflected GH's tendency to view themselves as becoming more attractive with age, whereas C viewed themselves as less attractive with age. Table 8 shows that for GH, height gain was positively correlated with all aspects of self-esteem for GH (*p* < 0.01), but for C, only taller adult height and Total Self Concept were correlated (*p* < 0.05).

### Academic Functioning

Although groups did not differ in their academic functioning (Table 7), both groups scored higher in reading than math, as is typical of this population (17, 18). Significant Session effects on the School scale (*p* < 0.001) and in Reading (*p* < 0.001) and Math (*p* < 0.05) reflected age-related improvements for both groups. Groups did not differ in grade failure or special-class placement (data not shown). In both groups, taller adult height was significantly (*p* < 0.05) associated with better school performance (Table 8).

## Discussion

### Overview of Current Study and Findings

It is well established that when patients with TS are supplemented with GH, they attain an average adult height of 5–7 cm above untreated patients but still remain short relative to unaffected peers. However, even though GH supplementation has been offered to TS patients now for more than two decades, its full impact on their psychosocial functioning has not been properly evaluated during or at trial completion (19). Given that patients with TS in the industrialized world are almost universally offered this treatment, obtaining a group of such patients to serve as untreated controls is unlikely. Consequently, our study of a wide range of psychosocial functions in patients with TS who received GH supplementation vs. those who did not are timely and fill the existing knowledge void.

Our findings are based on the majority of cases with TS who participated in a Canada-wide trial of GH (4) and were randomized to either a GH-supplementation or no-treatment control group. Both groups, and their parents, completed standardized questionnaires at set intervals until adult height was reached. Comparisons of treated and non-treated patients revealed remarkably few omnibus (i.e., across three post-baseline sessions) differences in their social skills, behavioral problems, self-esteem, or school functions. Nevertheless, they both scored quite poorly relative to the general population on most social-functioning indices and also showed moderately increased behavior problems. Furthermore, for many of the indices, their scores underwent significant changes over time with some characteristics, such as social competence and social relations, showing declines and others, such as behavior problems, improvement. Moreover, findings of significant Group X Session interactions for social problems, perceived popularity, withdrawn and anxious/depressed problems, and self-esteem reflected group differences in their patterns of change over time. Specifically, GH showed early benefits of therapy but C later caught up to them at the final-height session. When results were analyzed within individual sessions, results showed that just prior to puberty induction (session 2), GH claimed to be more socially engaged and popular than C and 1 year later (session 3), to show better behavioral adaptation and higher self-esteem than C. By contrast, C reported better social relations at session 3 and, according to parents, less anxiety and depression at adult height (session 4). These results, therefore, suggest the early benefits of GH therapy may diminish over time.

Of note, too, were our findings from single items showing C had generally more friends than GH but groups did not differ in time spent with friends or reported loneliness. However, C was teased more than GH, a factor known to contribute to increased depression and poor self-esteem in this population (20, 21). We studied this effect further by performing supplementary regression analyses in which we examined the relative contributions of height vs. teasing on anxiety/depression and self-esteem scores. As shown in Table 9, results indicated teasing (likely due to short stature and other physical stigmata) had a worse effect on self-esteem and depression than short stature itself, thus highlighting the need for additional therapies to counteract these adverse effects.

**Table 9:** Summary of simple regression analyses for variables predicting self- or parent-reported anxiety/depression and popularity scores in GH and C.

**Variable**	**GH Group**				**C Group**			
	***B***	***SE B***	**β**	***t***	***B***	***SE B***	**β**	***t***
**ANXIETY/DEPRESSION**^****a****^								
Height gain	−0.102	0.071	−0.181	−0.14	0.104	0.132	0.108	0.79
Adult height	0.071	0.717	0.012	0.10	−1.17	1.070	−0.152	−1.09
Teasing^a^	2.399	0.927	0.304	2.59^**^	−1.18	1.130	−0.094	−0.69
*R^2^*	0.139				0.034			
*F*	3.45^*^				0.615			
**ANXIETY/DEPRESSION**^**b**^								
Height gain	−0.101	0.095	−0.136	−1.07	−0.019	0.040	−0.045	−0.48
Adult height	0.579	0.992	0.077	0.58	0.618	0.322	0.181	1.92
Teasing^b^	2.830	1.321	0.264	2.15^*^	5.103	0.649	0.737	7.86^***^
*R^2^*	0.083				0.533			
*F*	1.94				20.94^***^			
**PHCSCS POPULARITY**^**a**^								
Height gain	0.655	0.467	0.180	1.40	−0.190	0.332	−0.069	−0.57
Adult height	1.597	4.465	0.045	0.350	3.605	2.705	0.162	1.33
Teasing^a^	−18.42	5.18	−0.42	−3.56^***^	−15.43	4.290	−0.433	3.59^***^
*R^2^*	0.248				0.238			
*F*	6.06^***^				5.63^**^			

a Based on YSR questionnaire;

b Based on CBCL questionnaire;

*** p < 0.001;

** p < 0.010;

* *p < 0.05*.

In addition, we found striking group differences in how adult-height indices were related to psychosocial outcome, with far fewer significant correlations for C than GH (2 vs. 21, χ^2^ = 18.24, *p* < 0.001). In C, only adult height and total self-concept and school functioning were correlated, whereas in GH, a taller adult height and/or greater height gain were associated with better parent- and/or self-reported social competence and social relations, more time spent with friends, higher self-esteem, and better school functioning, as well as less teasing, less loneliness, and fewer withdrawn or anxious/depressed behavior problems. The lattermost findings, which are displayed in Figure 5, indicate that the patients who grew more following GH therapy were subsequently less anxious, or conversely, *those who grew minimally had the most anxiety*. Relevantly, since items comprising both CBCL and YSR Anxiety/Depression scales reflect fearful behaviors, sadness, and worrying, as well as suicide contemplation, this may warrant future investigation to determine if those with the least growth are at increased risk.

### Findings From Uncontrolled Studies

The findings from this sub-study are at odds with the published literature on GH effects in TS, which is based mostly on uncontrolled trials where adult patients were assessed for psychosocial functioning via HRQoL questionnaires provided several years after competing therapy. For example, a nationwide study of French women with TS treated with GH showed similar HRQoL scores to the general female population. Results, which were unrelated to height (22) or other variables associated with GH treatment (e.g., duration of treatment) (22), were associated with other TS-related problems such as hearing disorders (23). This suggests that health problems and life events *other than short stature* may be contributing to the social impairments in patients with TS. In a Dutch study of women with TS aged ~20 years, who had participated in either a randomized dose-response trial from an early age or an open frequency-response trial from age 11 (12), results revealed reduced self-confidence and more psychosocial problems relative to a normative sample but no signs of depression (12). These same researchers also observed reduced social and emotional functioning relative to a reference population (24) on self- but not parent-report while scores were positively associated with breast satisfaction, but not height. A study from Belgium (25) of young TS women treated previously with GH and estrogen, who were assessed using similar instruments as in our sub-study, reported scores similar to a non-TS reference group at age 18–23 years. However, the treated TS patients had increased attention problems and reduced social acceptance while those with a 45, X karyotype also claimed greater than normal social withdrawal (25). It is important to emphasize that none of the above studies used a similarly followed untreated TS control group.

Two studies published more recently have involved expanded designs and a broader range of tests but are focused mainly on the effects of oxandrolone (O) therapy in TS. A study from Sweden (26) used a case-control design to compare four subgroups of young adult TS women and a population sample of similar aged women for multiple facets of quality-of-life. The TS patients were stratified according to whether or not they received O and/or GH; they were not randomly assigned to their respective conditions. A comparison of the 13 patients who were given only GH (i.e., no O) with the 34 without either GH or O (i.e., no treatment) revealed the GH-only group indicated less social isolation and less pain than the no treatment group. The second study, which was most like ours, used similar tests and a comparable time-frame for comparing groups (27). A total of 133 children with TS from 10 pediatric endocrine clinics across the Netherlands were randomized to three O conditions (placebo, low dose, high dose) with all receiving GH and estrogen; a no-GH comparison group was not studied. The three groups showed similarly elevated rates of internalizing problems and social withdrawal, which as in our study, decreased over the course of the study. Nevertheless, both of these studies are limited because group assignment was not random (26), the number of GH-only patients was small (26), and a no-treatment TS control group was not used (27). Thus, further study of the psychosocial consequences of GH therapy is needed.

### Explanations for Current Findings and Sources of Bias

It is not readily clear why the early psychosocial benefits of GH observed presently were not sustained, since the untreated group caught up to (or even surpassed) the treated group once adult height was attained. This finding supports the earlier report on a subset of our cohort who at age 20 indicated no benefits of GH supplementation on HRQoL (6). While some effects may have been eliminated had we used a double-blind placebo-controlled design, the one study with such a design reported no effects of GH supplementation on cognitive or academic functions after one to seven years; however, this study did not, to our knowledge, examine their social abilities (28).

A possible explanation for our findings is the GH group was initially biased to respond more favorably given their greater investment of time and effort with the hope that treatment would lead to favorable social outcomes (29). However, when after injections for at least 6 years and still being short relative to peers (despite slightly increased height), they and their parents may have become disillusioned or more realistic and so less biased in their responses. Another explanation why GH supplementation did not substantially improve psychosocial functioning at adult height may reflect the fact that short stature on its own has little consequence for psychological adaptation. According to Sandberg and Colsman (30), this effect becomes insignificant once other factors such as parents' education and marital status are taken into consideration. It is important to note, however, that when we reanalyzed our data using marital status and SES as covariates in a supplementary analysis (results not shown), most effects not only remained in the GH group, some such as social-relation difficulties became worse and others, such as self-concept and happiness, improved.

Alternatively, the improvements we observed in untreated control patients at adult height may have been real reflecting resignation to their height status and satisfaction with achieving puberty coincident with peers (24); notably, the natural course of TS development and the impact of estrogen therapy are not known (25). However, it is also possible the C cases, who completed our study, represented better functioning individuals than those who dropped out midway through the trial.

It is also not evident why C had more friends than GH throughout the trial, especially at session 4. Although C also had more friends at baseline, the difference from GH in number of friends was not significant at this session. Although it does not seem likely that the GH group was hindered in their friendships by the therapy, this factor needs further investigating.

Our findings that specific internalizing behavioral difficulties were associated with adult height status may reflect parents' misattributing their daughter's troubles to the most obvious possible culprit, namely her short stature, a phenomenon sometimes referred to as a “focusing illusion” (31). Previous findings of psychosocial impairment in patients with short stature may possibly have ignored the fact that their short stature was part of a more serious medical condition. The emotional burdens of these other medical co-morbidities are unfortunately not improved by GH supplementation.

In addition, it should be noted that while GH treatment was not found to be particularly effective in improving psychological well-being from the non-treated state, current findings of higher than normal rates of psychosocial problems among TS patients, as observed by others (32), cannot be ignored. For example, all TS patients showed significantly reduced social competence and social relations scores and had elevated social and behavioral problem scores. It is interesting to note, however, that our patients scored somewhat more favorably in terms of BP than those from the Netherlands receiving oxandrolone in combination with GH (27).

Moreover, it is not readily clear why there were so few correlations between height and outcome in the untreated C group. It is possible that this reflects the computation of correlations on absolute height measures and not adjusting height for parents' heights. For example, some shorter parents may have been more accepting of their daughter's short stature than taller parents and this may have contributed to more self-confidence and better social adjustment among those from shorter parents. However, further analysis adjusting for target height was unfortunately not possible because not enough parents were measured for height in our study. In contrast, the large series of significant correlations in the treated group may be that new expectations (and hope) from therapy overrode these initial within-family effects.

The current findings, therefore attest to the possibility of negative consequences from unmet expectations, which need to be addressed by therapies beyond just hormones. Indeed, in a recent review paper on the care of girls and women with TS, Culen et al. (33) claim it is important to provide these patients with state-of-the-art psychosocial therapies after beginning GH treatment. Patients with TS need treatments for coping with the psychosocial challenges of their condition (34), such as counseling (33) and social skills training (35), as well as therapy targeted toward maintaining self-esteem in the face of the negative emotional consequences that accompany the physical and health challenges of this condition (36). Therapies also need to deal with the impact of the teasing, as well as the bullying they may receive (34). It is also important to monitor other TS-related problems such as hearing disorders (22), since minimizing these symptoms may lead to improved quality of life. Given our findings that the TS patients with the poorest GH response were at increased risk of further psychological problems, specific additional resources need to be in place for this subgroup of patients.

### Limitations

According to Gardner et al. (37), all studies of GH therapy for short stature (of any cause), including randomized trials, suffer varying degrees of bias. Particular sources of bias include: (i) sequence generation, or how subjects are assigned to different groups despite randomization, (ii) allocation concealment, (iii) blinding, (iv) co-interventions, and (v) selective reporting and data loss. Although our study suffers a few of these shortcomings, it should be pointed out that because randomization was based on stratification for initial height, this source of bias was minimized to a degree. Unfortunately, blinding could not be achieved for children, parents, and medical staff, who all knew the patient's group assignment, since we had a no-injection, not a placebo-injection, control group; however, blinding was maintained for personnel involved in scoring questionnaires and inputting data. We could not control for co-intervention with estradiol, which was given to more than 90% of our sample and since we did not receive information on who were or not additionally treated in this way, we could not control for this in the data analysis. Because puberty induction at a normal age is essential for adult well-being (24), this too may have confounded GH benefits. Furthermore, information was also lacking on treatment adherence and protocol compliance. Regarding selective reporting, all data collected from the sub-study are presented in this report, except those from a family functioning questionnaire because its unusual scoring method was not adaptable for our data-analytic approach and we did not consider it a valid outcome measure for our objectives. Of note, the rate of data loss for this sub-study was relatively low compared with other psychosocial studies of GH effects (38). Of concern was a factor out of our control, namely the differential but non-significant loss in the control group at the third session due to provision of GH by a private physician. As well, we lacked baseline data on a small set of patients from a clinic where the staff endocrinologist did not initially want to participate but agreed later. It should be noted that we used approved imputation techniques for replacing missing data (13) and were careful to correct for multiple comparisons on instruments providing manifold subscales.

Several methodological limitations of our study also warrant further discussion. The first is we may have lacked sufficient power to find significance with the sample sizes available to us since the original study was powered for detecting height differences between treated and untreated groups. Using existing power tables (15), we found that with ~65 cases per group, we could at best detect moderate sized effects. When we deployed the established literature on TS and GH to identify what are the effect sizes in these studies (which demonstrated a high degree of variability among themselves), we noted moderate-sized effects for some abilities such as social functions but small effect sizes for others, such as behavioral issues. This signifies the need for larger samples than what were available to us. Also, computation of the power associated with the three trend-level group X session interactions (i.e., CBCL Social Problems, YSR Total Social Competence, PHCSCS Physical Appearance) revealed β-levels of 0.54, 0.51, and 0.56 respectively, indicating our risk of missing true effects was elevated.

A second methodological limitation concerns the tools we used to evaluate psychosocial outcome, which did not directly examine cognitive abilities that may also be sensitive to GH effects (5, 38). Moreover, given the multicenter nature of our study, we had to rely upon questionnaires, which may not have been sensitive enough to evaluate subtle effects arising from GH therapy. Furthermore, several superior social functioning measures became available after our study began. One, for example, the Social Responsiveness Scale, has been shown to strongly discriminate between TS and non-TS controls (32) and was recommended for a TS assessment battery (33). Thirdly, our study was conducted in two languages (i.e., English and French), that may have increased variability. However, it should be noted that all instruments and instructions were professionally translated, no differential loss was noticed between Anglophone vs. Francophone sites, and randomization was equivalent across English and French sites.

Notably, too, we lacked several key pieces of information, which also may have influenced our results. First, we were not provided the information on the patients' karyotype, which too could have influenced both the growth response and psychosocial outcome. Regrettably, too, the heights of parents were not available for determining target height and analyzing effects of the psychosocial intervention. It is noteworthy that in a subsequent observational study at one of the participating sites, parental height did not differ between those choosing GH-therapy vs. no GH (39).

## Conclusions

The equivocal results of the present study reflecting only modest gains in psychosocial functioning among patients with TS treated with GH should not deny them the option of GH supplementation, particularly as more favorable psychosocial outcome on a number of indices was strongly associated with ultimate height or height gain. Furthermore, their increased stature has the potential to improve their abilities to better adapt to their physical environment (e.g., driving, occupational opportunities etc.), which can later lead to improved quality of life (35). It is important to note that when girls with TS and their families from one participating center were presented with the available evidence, the vast majority (78%) chose the option of GH supplementation (39). Since it is possible that starting treatment at an earlier age may lead to a more discernible psychosocial benefit, given our findings of taller height as well as greater height gain in those who started treatment younger, this possibility needs to be explored in future studies of psychosocial outcome.

When counseling these patients and their families, current results suggest that it is important not to overemphasize the benefits of GH supplementation on psychosocial adaptation due to increased height, especially when response to treatment for some patients may be minimal. Above all, expectations should always be kept realistic and all TS-associated health problems should be addressed.

## Data Availability

The datasets generated for this study are available on request to the corresponding author.

## Author Contributions

JR spearheaded the psychosocial component under the initial guidance of Dr. Jack Holland. She oversaw all activities including packet preparation and distribution to the many sites and followed their return; she managed the activities of the research assistants in data collection, database scoring, and preliminary statistical analyses; she conducted the final statistical analysis and wrote or co-wrote all earlier versions of the manuscript. GVV gave direction and impetus to manuscript preparation and co-wrote an earlier and the current version of this manuscript. He has been an international spokesperson for this trial, which he entered after its initiation once he immigrated to Canada. He also headed the site at Hôpital St. Justine.

### Conflict of Interest Statement

The authors declare that the research was conducted in the absence of any commercial or financial relationships that could be construed as a potential conflict of interest.
