# Supplementary material for: Growth Hormone Supplementation and Psychosocial Functioning to Adult Height in Turner Syndrome: A Questionnaire Study of Participants in the Canadian Randomized Trial
Source: Front Endocrinol (Lausanne). 2019 Mar 13;10:125. doi: 10.3389/fendo.2019.00125 (PMC6425861; doi:10.3389/fendo.2019.00125)
Supplement: Supplementary file 1 [file Table_1.DOCX]

**SUPPLEMENTARY TABLE 1**. List of Participating Institutions with Numbers of Patients Enrolled at Each

| **Hospital/Institution** | **City** | **N** |
| --- | --- | --- |
| Alberta Children’s Hospital | Calgary | 13 |
| Edmonton University Hospital | Edmonton | 10 |
| Izaak Walton Killam Hospital | Halifax | 9^a^ |
| McMaster University | Hamilton | 3 |
| Children’s Hospital Western Ontario | London | 6^b^ |
| Hôpital St. Justine | Montreal | 9 |
| Montreal Children’s Hospital | Montreal | 5 |
| Children’s Hospital Eastern Ontario | Ottawa | 9 |
| Children’s Hospital Université de Laval | Quebec City | 16 |
| Children’s Hospital Université de Sherbrooke | Sherbrooke | 7 |
| The Hospital for Sick Children | Toronto | 26^c^ |
| BC Children’s Hospital | Vancouver | 14 |
| Children’s Hospital of Winnipeg | Winnipeg | 5 |

Note: Relocated during trial to ^a^Vancouver, ^b^Ottawa, ^b^London
