# Supplementary material for: Growth Hormone Supplementation and Psychosocial Functioning to Adult Height in Turner Syndrome: A Questionnaire Study of Participants in the Canadian Randomized Trial
Source: Front Endocrinol (Lausanne). 2019 Mar 13;10:125. doi: 10.3389/fendo.2019.00125 (PMC6425861; doi:10.3389/fendo.2019.00125)
Supplement: Supplementary file 2 [file Table_2.DOCX]

**SUPPLEMENTARY TABLE 2**. Mean (SE) CBCL Results at Session 1 (Baseline)

|  |  | | | | |  | | |  |  |  |
| --- | --- | --- | --- | --- | --- | --- | --- | --- | --- | --- | --- |
|  |  | | | | **GH** | | | **C** | |  |  |
| **Social Competence**^a^ | |  | |  |  |  |  |  |  |  |  |
| Total Social Competence | |  | -0.70 (0.10) | | | | -0.78 (0.09) | | | |  |
| Activities | |  | -0.49 (0.07) | | | | -0.63 (0.10) | | | |  |
| Social Relations | |  | -0.56 (0.09) | | | | -0.70 (0.09) | | | |  |
| Social Problems | |  | 1.24 (0.12) | | | | 1.31 (0.13) | | | |  |
| Number of friends^ç^ | |  | 2.96 (0.10) | | | | 3.16 (0.10) | | |  | |
| Time with friends^c^ | |  | 2.12 (0.09) | | | | 2.22 (0.09) | | | |  |
| Teased^d^ | |  | 0.87 (0.09) | | | | 0.91 (0.09) | | | |  |
| **Behavior Problems**^b^ | |  |  | | | |  | | |  | |
| Total Problems | |  | 0.68 (0.07) | | | | 0.75 (0.07) | | |  | |
| Internalizing Problems | |  | 0.59 (0.06) | | | | 0.65 (0.09) | | |  | |
| Externalizing Problems | |  | 0.41 (0.07) | | | | 0.47 (0.08) | | |  | |
| Withdrawn | |  | 0.67 (0.09) | | | | 0.65 (0.09) | | |  | |
| Anxious/depressed | |  | 0.60 (0.08) | | | | 0.76 (0.10) | | |  | |
| Thought Problems | |  | 0.55 (0.08) | | | | 0.62 (0.08) | | |  | |
| Attention Problems | |  | 1.06 (0.12) | | | | 1.14 (0.12) | | |  | |
| Delinquency | |  | 0.40 (0.07) | | | | 0.43 (0.08) | | |  | |
| Aggression | |  | 0.41 (0.08) | | | | 0.55 (0.10) | | |  | |
| **School Functioning** | |  |  | | | |  | | | |  |
| School^a^ | |  | -0.85 (0.10) | | | | -0.72 (0.12) | | | |  |
| Reading^e^ | |  | 3.33 (0.02) | | | | 3.19 (0.08) | | | |  |
| Math^e^ | |  | 2.70 (0.08) | | | | 2.86 (0.09) | | | |  |

^a^Expressed in SD units with lower negative scores signifying better social functioning; ^b^Expressed in SD units with higher positive scores signifying more problems; ^ç^Scored on a 4-point scale (1 = “none”; 2 = “1”; 3 = “2 or 3”; 4= “4 or more”); ^d^Scored on a 3-point scale (1 = “not true”; 2 = “somewhat or sometimes true”; 3 = “very true or often true”); ^e^Based on a 4-point scale (4=very good)
