# Supplementary material for: Growth Hormone Supplementation and Psychosocial Functioning to Adult Height in Turner Syndrome: A Questionnaire Study of Participants in the Canadian Randomized Trial
Source: Front Endocrinol (Lausanne). 2019 Mar 13;10:125. doi: 10.3389/fendo.2019.00125 (PMC6425861; doi:10.3389/fendo.2019.00125)
Supplement: Supplementary file 3 [file Table_3.DOCX]

**SUPPLEMENTARY TABLE 3**. Mean (SE) Session 1 (Baseline) Scores from Piers-Harris Children’s Self-Concept Scale^a^

|  |  | |  | | | | |  | | | |  |
| --- | --- | --- | --- | --- | --- | --- | --- | --- | --- | --- | --- | --- |
|  |  | | | | | **GH** | **C** | | | | |  |
| Total Self Concept | |  | | | 71.5 (2.7) | | | | 68.0 (2.8) | | | |
| Behavioral Adaptation | |  | | *77.3 (2.2)* | | | | | *67.8 (3.4)* |  |  |  |
| Intellectual & School Status | |  | | 67.5 (2.7) | | | | | 69.2 (3.1) |  |  |  |
| Physical Appearance | |  | | 57.9 (3.0) | | | | | 60.5 (3.0) |  |  |  |
| Freedom from Anxiety | |  | | 61.4 (3.1) | | | | | 58.6 (3.4) |  |  |  |
| Popularity | |  | | 49.9 (3.0) | | | | | 44.1 (3.7) |  |  |  |
| Happiness/satisfaction | |  | | | 67.0 (3.0) | | | | 72.5 (2.5) | |  |  |

^a^Expressed in percentile scores; Results shown in italics indicate significant group difference at the *p*<0.05 level
